# Royal jelly-like protein localization reveals differences in hypopharyngeal glands buildup and conserved expression pattern in brains of bumblebees and honeybees

**DOI:** 10.1242/bio.20147211

**Published:** 2014-03-25

**Authors:** Štefan Albert, Johannes Spaethe, Kornelia Grübel, Wolfgang Rössler

**Affiliations:** Department of Behavioral Physiology and Sociobiology, Biocenter, University of Würzburg, Am Hubland, D-97074 Würzburg, Germany

**Keywords:** Bumblebee, *Bombus*, Brain, Hypopharyngeal glands, Labial glands, Immunohistochemistry, Kenyon cells, Mushroom bodies, Honeybee

## Abstract

Royal jelly proteins (MRJPs) of the honeybee bear several open questions. One of them is their expression in tissues other than the hypopharyngeal glands (HGs), the site of royal jelly production. The sole MRJP-like gene of the bumblebee, *Bombus terrestris* (BtRJPL), represents a pre-diversification stage of the *MRJP* gene evolution in bees. Here we investigate the expression of *BtRJPL* in the HGs and the brain of bumblebees. Comparison of the HGs of bumblebees and honeybees revealed striking differences in their morphology with respect to sex- and caste-specific appearance, number of cells per acinus, and filamentous actin (F-actin) rings. At the cellular level, we found a temporary F-actin-covered meshwork in the secretory cells, which suggests a role for actin in the biogenesis of the end apparatus in HGs. Using immunohistochemical localization, we show that *BtRJPL* is expressed in the bumblebee brain, predominantly in the Kenyon cells of the mushroom bodies, the site of sensory integration in insects, and in the optic lobes. Our data suggest that a dual gland-brain function preceded the multiplication of *MRJPs* in the honeybee lineage. In the course of the honeybee evolution, HGs dramatically changed their morphology in order to serve a food-producing function.

## INTRODUCTION

Glands serve manifold functions in insects ranging from reproduction, communication and food processing to defense and nest building (Chapman, 2012). Hypopharyngeal glands (HGs) are specific to Hymenoptera (Cruz-Landim, 1998). They are paired secretory organs usually located bilaterally in the frontal head region entering in the suboral plate of the hypopharynx (Snodgrass, 1956). It was hypothesized that the original function of HGs in food digestion and modification has been modified in the course of evolution into a nutritive, food-secreting function in honeybees, which was accompanied by their growth (Kupke et al., 2012).

Hypopharyngeal glands are extremely variable in size and morphology across and within species. Usually, the secretory acini are connected by short necks with a collecting duct of variable length. In honeybee workers, for example, the extended HGs may reach the length of the entire body (own observations). In wasps, HGs consist of secretory acini individually connected to the hypopharyngeal plate (Britto and Caetano, 2006). Distinct differences in HG size and morphology have also been reported between sexes. In some stingless bees (Meliponini), HGs are present only in the female caste, in others both females and males possess HGs (Costa and Cruz-Landim, 1999). Honeybee nurses possess large HGs, which enlarge their volume until about day 10 after adult eclosion and shrink after the onset of foraging (>day 15 (Deseyn and Billen, 2005)). HGs of honeybee queens and drones are vestigial (Snodgrass, 1956). In contrast to honeybees, both female castes in bumblebees (*Bombus*) possess HGs. In contrast to honeybee, HGs of bumblebee queens are even larger than those of workers (Kupke et al., 2012). Controversial data were published about the presence of HGs in bumblebee drones (Palm, 1949; Svensson and Bergström, 1977; Terzo et al., 2007).

Major royal jelly proteins (MRJPs) make up a subfamily of closely related proteins belonging to a superfamily of Yellow/MRJP proteins (Drapeau et al., 2006; Ferguson et al., 2011). These proteins were named after their initial identification as a dominant component of honeybee RJ (Schmitzová et al., 1998), which is produced in the HGs. Due to their absence in the genomes of other insects, MRJPs were thought to be diversified only in the genus *Apis* and became a major component of the RJ (Albert and Klaudiny, 2004; Drapeau et al., 2006). However, identification of MRJP homologs in other Hymenoptera questioned this evolutionary scenario (Smith et al., 2011; Werren et al., 2010). Moreover, advances in honeybee biochemistry and neuroanatomy revealed that MRJPs are also expressed outside the HGs, the major site of MRJP production: two MRJPs, MRJP8 and MRJP9, were found in the honeybee venom (Blank et al., 2012; de Graaf et al., 2009; Peiren et al., 2008), and several others in different parts of the brain (Hernández et al., 2012; Hojo et al., 2010; Kucharski et al., 1998). MRJP1, but none of the other four tested MRJPs, was found to play a central role in the queen–worker polymorphism, in particular in determining the development of honeybee queens (Kamakura, 2011). Apparently, functional diversification and specialization have accompanied multiplications of *MRJP* genes in the course of evolution (Albert et al., 1999b), but it remained unclear, which function can be assigned as the plesiomorphic one. Recently, we found that the bumblebee genome contains only a single *MRJP*-like gene, which was suggested to represent a pre-multiplication state of the MRJP evolution in Apidae (Kupke et al., 2012). The gene, termed *BtRJPL* (***B****ombus ****t****errestris* **r**oyal **j**elly **p**rotein-**l**ike), was shown to share many features with *MRJPs* of honeybees. Furthermore, it is expressed mainly in the hypopharyngeal glands, even though bumblebees do not produce larval food similar to the royal jelly in honeybees. We proposed that the digestive/food modifying function was the most likely original function of the MRJP protein before multiplication of its gene and adaptation of a novel nutritive function took place (Kupke et al., 2012). However, whether BtRJPL is also expressed elsewhere from the HGs, like it was shown in the honeybee, is unknown.

Here we investigated the morphology of the HG in bumblebee males and females and the expression of the BtRJPL protein in the HGs and the brain by means of immunohistochemistry.

## MATERIALS AND METHODS

Honeybees, *A. mellifera*, were collected from the apiary of the University Würzburg. Bumblebees, *B. terrestris*, were purchased from Koppert (Berkel en Rodenrijs, Netherlands) and kept in an air-controlled room at constant 60% humidity and 25°C temperature and a 12/12 hours day/night regime. To collect individuals of defined age, freshly eclosed animals were captured, marked individually with a plastic tag on their thoraces and put back into the colony.

### SDS-PAGE and immunoblotting

Dissected glands or brains were homogenized in 100 µl of SDS-PAGE loading buffer and boiled for 5 min. Appropriate amounts of extracts (between 0.2 and 6.0 brain and HG equivalent, respectively) were loaded on a vertical 10% SDS-PAGE gel and electrophoresed at 15 V/cm (horizontal gel system, PeqLab, Erlangen, Germany). Obtained gels were either stained with colloidal Coomassie blue G-250 (Sigma, St Louis, USA) or blotted onto nitrocellulose membrane (semidry system, 2 V/cm^2^, PeqLab). Blotting membranes were blocked overnight with 5% skimmed milk in Tris-buffered saline with Tween 20 TBST (10 mM Tris, pH = 7.4, 150 mM NaCl, 0.05% Tween 20). Primary antibodies were diluted in TBST as follows: rabbit affinity-purified α-BtRJPL (Kupke et al., 2012) 1:1,000, and goat α-actin (Santa Cruz, San Diego, USA) 1:500. Incubation varied from 3 hours to overnight. After washing 4×10 min with TBST, the blots were incubated for 1 hour with fluorescence-labeled secondary antibodies (anti-goat 680 and anti-rabbit 800; LI-COR Biosciences, USA) diluted 1:20,000 in TBST. After final washing 4×10 min with TBST, immunoreactive bands were detected by an Odyssey infrared imaging system (LI-COR Biosciences, USA).

### Immunohistochemistry

Glands and brains were dissected under a stereo microscope (Wild M3C, Leica Wetzlar, Germany) and fixed in ice-cold 4% formaldehyde in phosphate buffered saline (PBS) overnight. After washing 3×10 min in fresh PBS, the tissues were embedded in 5% LMP agarose (Amresco, Solon, USA), and 100 µm sections were prepared using a vibrating microtome (Leica VT 1000S, Nussloch, Germany).

Sections were washed with 2% Triton X-100 in PBS, then 0.2% Triton X-100 in PBS and pre-incubated with 2% normal goat serum (NGS, Dianova, Hamburg, Germany) in PBS + 0.2% Triton X-100 (PNGT). Afterwards the sections were incubated with affinity-purified rabbit antibodies against BtRJPL (Kupke et al., 2012), diluted 1:50 in PNGT buffer for two days at 4°C. After washing 5×10 min with PBS the sections were incubated with secondary Alexa 568-conjugated goat anti-rabbit serum (1:250) and CF633-conjugated phalloidin (Biotrend, Cologne, Germany), diluted 1:200 in PBS + 1% NGS overnight at 4°C. Next day, the samples were washed 2×10 min with PBS and incubated for 15 min with Hoechst 34580 (Molecular Probes, Leiden, The Netherlands) diluted 1:1,000 in PBS. After final washes for 4×10 min with PBS the samples were transferred into 60% glycerol in PBS, incubated for 30 min and mounted in 80% glycerol in PBS on slides. Sealed slides were stored at 4°C. Control specimens were treated identically with omission of primary anti-BtRJPL antibodies. All experiments were repeated at least five times.

### Laser-scanning confocal microscopy

Preparations of bee brains and glands were scanned at different magnifications using a laser-scanning confocal microscope (Leica TCS SP2, Leica Microsystems, Wetzlar, Germany). Image processing and F-actin ring diameter measurements were done using IMAGE-J software. Significance of measured differences was tested by Mann–Whitney U-test. For the reconstruction of the nuclear shape, 54 optical sections of 1 µm thickness were taken through the whole nucleus. Obtained stacks were used for reconstruction of 3-D shape using AMIRA software (Mercury Computer Systems, Berlin, Germany).

## RESULTS

### Honeybee MRJPs are recognized by anti-BtRJPL antibodies

**Fig. 1.**
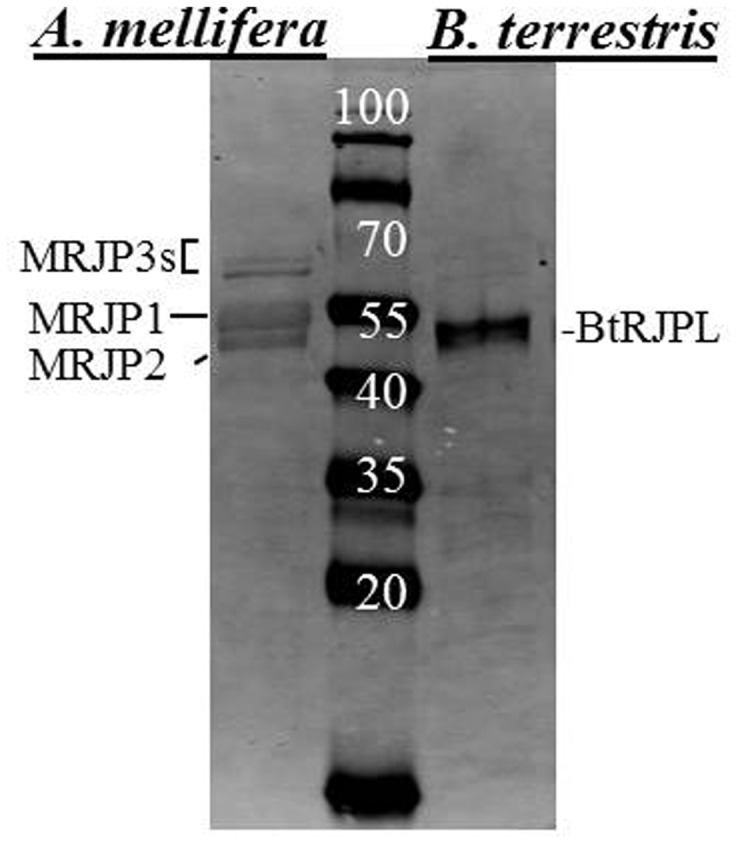
Western blotting analysis with α-BtRJPL antibodies. Western blotting analysis with α-BtRJPL antibodies shows that the antibody recognizes a single BtRJPL band in the HGs of bumblebees and multiple MRJP bands in the honeybee HGs, which were marked according to their electrophoretic mobility (Schmitzová et al., 1998).

In the bumblebee HGs, the antibody recognizes a polypeptide of 51–54 kDa, which is approximately the size found in immunoblots from head extracts of bumblebee queens and workers (Kupke et al., 2012). Due to the high similarity of MRJP and BtRJPL primary structures (69–73% by BLAST, with several blocks of amino acids that are completely identical (supplementary material Fig. S1)), we asked whether polyclonal antibodies raised against BtRJPL would also recognize honeybee MRJPs in HGs and RJ. To test this we prepared protein extracts of honey bee HGs in addition to the bumblebee HGs. Honeybee HGs are larger and have very high protein content. Therefore we took equal amounts of total proteins, electrophoresed them and tested the antibodies by immunoblotting. Immunoblot analysis confirmed the cross-reactivity of the BtRJPL-specific antibodies with at least MRJP1–3 proteins of the honeybee HGs (Fig. 1) (Albert et al., 1999a; Schmitzová et al., 1998).

### BtRJPL expression in secretory cells of the hypopharyngeal gland

**Fig. 2.**
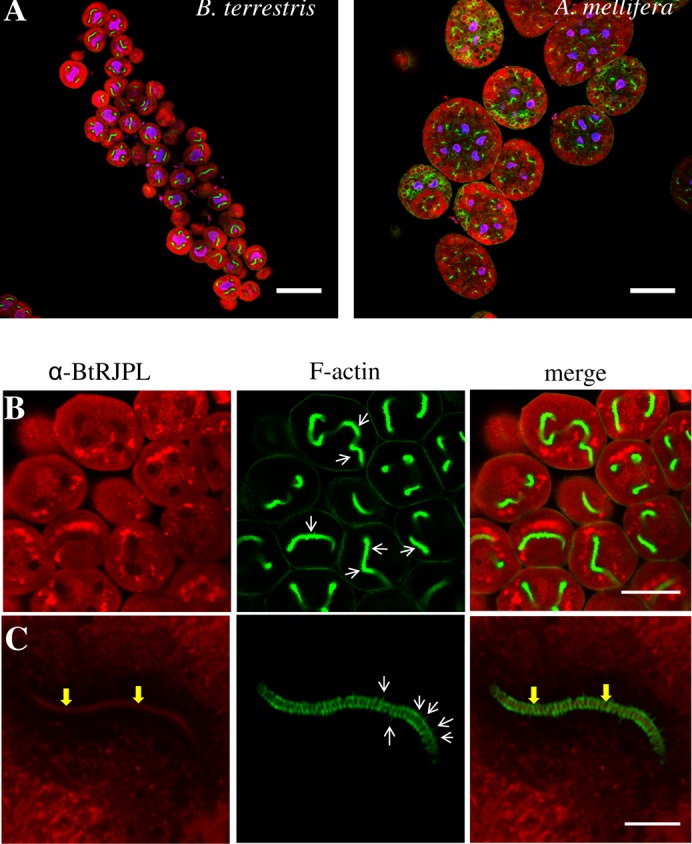
BtRJPL immunoreactivity in hypopharyngeal glands. (A) Comparison of bumblebee and honeybee hypopharyngeal glands. Dissected hypopharyngeal glands were treated with Hoechst stain (blue), fluorescently labeled phalloidin (green), α-BtRJPL antibodies (red). Multiple nuclei in honeybee HG (blue spots) indicate multicellular organization of secretory acini. Bumblebee acini are unicellular. Both pictures were taken at the same magnification. (B) Detailed view of the secretory cells of bumblebee hypopharyngeal glands. BtRJPL-ir is present in the cytosol and concentrated in the vesicular compartments. F-actin forms conspicuous tubes in the cytosol of secretory cells (green). These structures were previously termed end apparatus (Noirot and Quennedey, 1974). (C) Detailed view of the end apparatus of a secretory cell. The tube consists of densely packed actin rings (green). The BtRJPL signal in the lumen of the end apparatus, which transports secretion towards secretory ductus (arrows), confirms that the protein is secreted by the secretory cells. White arrows in the middle panels of B and C point to actin spikes (see main text). Scale bars: 100 µm (A), 50 µm (B), 10 µm (C).

Using RT-qPCR and immunoblotting, HGs were previously shown to express BtRJPL (Kupke et al., 2012). Applying immunohistochemistry for detection of BtRJPL-ir in HGs, an intensive cytosolic labeling of HG secretory cells in both bumblebees and honeybees was observed (Fig. 2A). For orientation, F-actin was stained with fluorescently labeled Phalloidin. Besides the well known cytoplasmic membrane-localized cortical actin, it also formed conspicuous tubular structures in the cytosol of bumblebee secretory cells (Fig. 2B). Cytosolic BtRJPL signal was concentrated in globular, possibly membrane-enclosed structures of different sizes that were often stacked near the F-actin tubes. In some cases BtRJPL signal could clearly be identified in the lumen of the F-actin tubes (Fig. 2C). Interestingly, BtRJPL signal intensity was weaker in the region surrounding the actin tubes (Fig. 2A,C; supplementary material Fig. S2). The localization of the BtRJPL signal in immunostainings gives strong support to our previous assumption that this protein appears to be secreted by the HGs (Kupke et al., 2012).

### Hypopharyngeal glands are present in bumblebee males and females

**Fig. 3.**
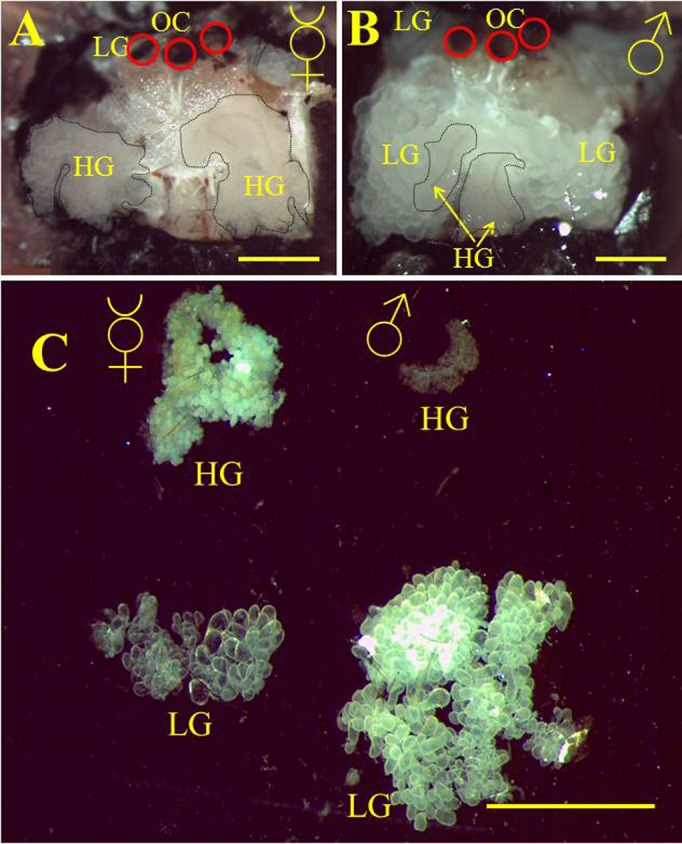
Hypopharyngeal glands in bumblebee males. Frontal cuticle was removed from the heads of *B. terrestris* worker (A) and drone (B). The underlying space was mainly filled with labial glands (LG) with large acini in males and with hypopharyngeal glands (HG) in females. In the central part of the male head the distinct glandular tissue of HGs with brownish duct and small acini could be observed (dashed lines). Hypopharyngeal glands were uncovered from the overlaying LG tissue by forceps. Red circles in both pictures indicate the positions of ocelli (OC). (C) Dissected head glands of a worker (left) and a drone (right). Labial glands (LGs) fill the posterior space of the head in both males and females. Anterior part of the male's head is filled by large LGs hiding small HGs. Scale bars: 1 mm (A,B), 2 mm (C).

**Fig. 4.**
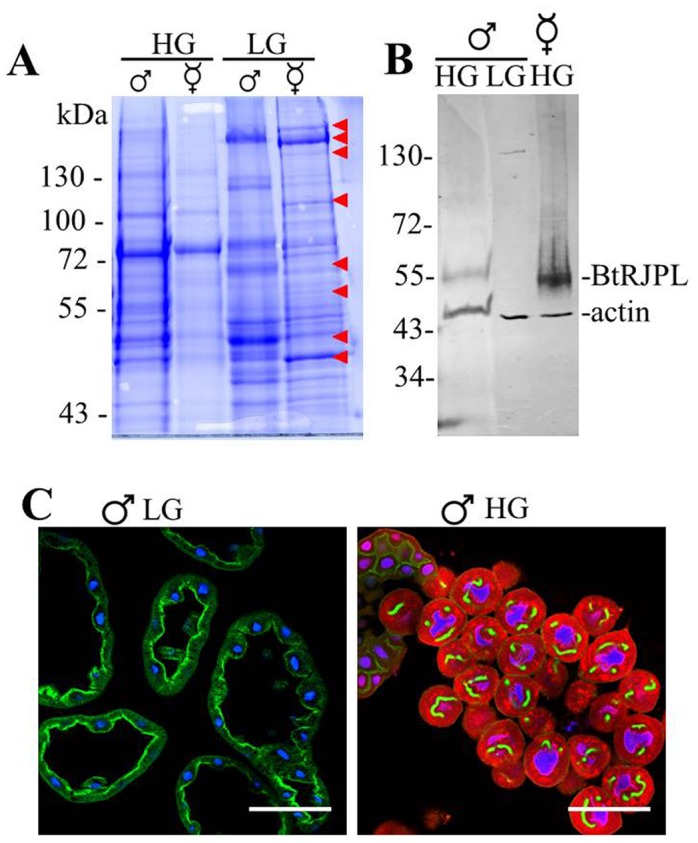
Biochemical and immunohistochemical characterization of the *B. terrestris* male head glands and their comparison with head glands of females. (A) Protein profiles. Proteins of hypopharyngeal and labial glands were size-separated by SDS-PAGE and stained with Coomassie blue. Protein profiles of male and female HGs are similar, those of LGs show sex-specific differences. Red arrowheads point to some of the protein bands differing between male and female LGs. Observed differences suggest different functions of male and female LGs. (B) BtRJPL is express in male HGs but absent in LGs. Actin (as a control protein) was detected by immunoblotting of size-separated protein extracts of both HGs and LGs. Male HGs extract is a pool of six HGs. Female HGs extract represents 20% of total HGs of a single worker. (C) Histology of male LG (left) and HG (right) secretory acini. LG acini are build-up of multiple cells forming an epithelial sac, the acini of HG are unicellular. Blue: nuclei (Hoechst), green: phalloidin-labeled F-actin, red: BtRJPL. Scale bars: 100 µm.

As mentioned above, we found that in contrast to the honeybee, both female castes in bumblebees possess HGs. HGs in queens are even larger than in workers. Previous reports on the presence of HGs in bumblebee males were inconsistent. Male HGs were mentioned by Palm (Palm, 1949), but without a detailed description, and HGs were not mentioned at all in a number of other publications describing the cephalic glands of bumblebee males (Svensson and Bergström, 1977; Terzo et al., 2007). To re-investigate the presence/absence of HGs in the light of our findings on BtRJPL localization, we dissected heads of *B. terrestris* females (Fig. 3A) and males (Fig. 3B). As described previously (Svensson and Bergström, 1977; Terzo et al., 2007), and in contrast to females, the frontal area of the drone heads was filled with the glandular tissue of labial glands (LGs) containing large acini (Fig. 3B). In addition we found in the medial frontal region an additional pair of glands with much smaller acini attached to a brownish duct (Fig. 3A,B, dashed lines). These glands opened into the mandibular plate. Thus the morphology and location of these putative male HGs resemble that of female HGs, but at a much smaller size (Fig. 3C). We hypothesized that, if this glandular tissue differs from the LGs (and is probably part of the HGs), it should express different proteins than the LGs, which are assumed to produce the male sex pheromone (Terzo et al., 2007). We size-separated proteins of male LGs and the putative HGs by means of SDS-PAGE and compared the protein pattern with that of the worker HGs and LGs. The protein profiles of male HGs and LGs differed significantly (Fig. 4A, lanes marked with HG and LG). On the other hand, the protein profiles of female and putative male HGs appeared rather similar (Fig. 4A). Finally, the protein profiles of male and female LGs differed substantially, indicating different roles in the physiology of males and females. HG-specific expression of BtRJPL, in female and male putative HGs was confirmed by immunoblotting (Fig. 4B). Confocal microscopy investigations (Fig. 4C) revealed that male HGs are formed by single secretory cells, similar as it is known from female HGs, whereas LGs are formed by a layer of epithelial cells.

Taken together, our results strongly suggest that *B. terrestris* drones possess small but distinct HGs. These glands are located more centrally compared to females, often localized beneath the large LGs. The LGs and HGs in males and females differ morphologically at both macro- and microscopic levels, and they produce different and distinct subsets of proteins.

### Comparison of the bumblebee and honeybee HG secretory cells

Secretory cells of the bumblebee HGs exhibited several distinct features. Each acinus of *B. terrestris* HGs is formed by a single secretory cell (diameter ∼60 µm; Figs 2, 4). In contrast, in the honeybee HGs acini are visibly larger (diameter ∼200 µm), consisting of at least 8 cells (8 nuclei could be identified in a single focus layer of one acinus; Fig. 2A). In both species secretory cells contained long convoluted filamentous actin (F-actin)-decorated tubular structures (Fig. 2A) connected to an extracellular ductus (not shown). According to the classification of Noirot and Quennedey, these cells belong to class 3 insect secretory cells (Noirot and Quennedey, 1974) and the intracellular tubes have been termed ‘end apparatus’ (EA).

**Fig. 5.**
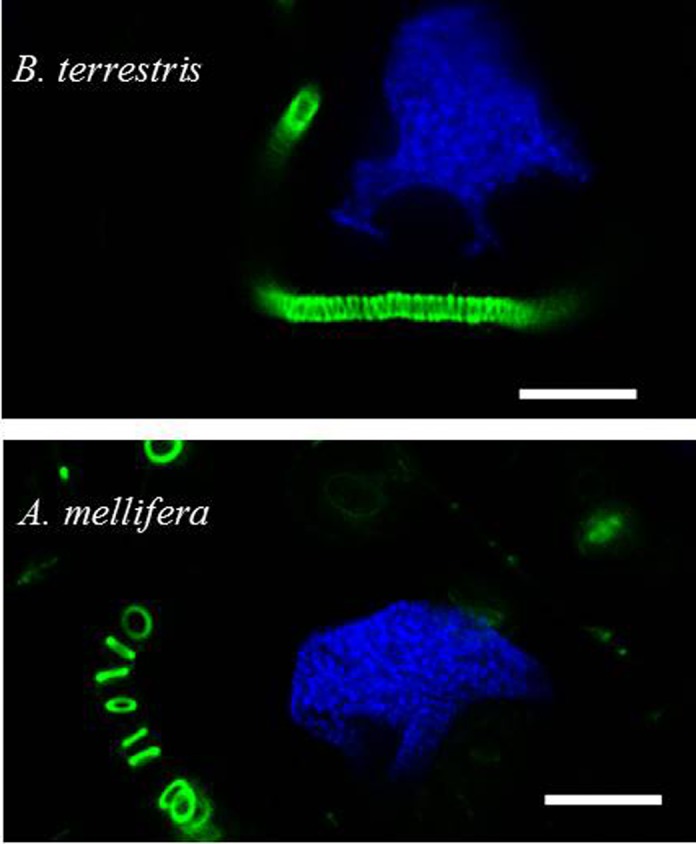
F-actin rings of bumblebee and honeybee secretory cells. Dissected hypopharyngeal glands were treated with Hoechst stain (blue) and fluorescently labeled phalloidin (green). Actin rings of the bumblebee's end apparatus are smaller in diameter (∼2.0 µm), densely stacked, and slightly leaned to each other; those of honeybees have a larger diameter (∼3.1 µm) and do not contact each other. Scale bars: 10 µm.

In *B. terrestris*, rings formed by F-actin were tightly stacked to form a nearly contiguous tube (Fig. 5A). In the honeybee, individual rings were regularly but more loosely distributed along the EA (Fig. 5B). Interestingly, in some bumblebee individuals we observed actin “spikes” protruding from the rings towards cytosol (Fig. 2B,C, white arrows). These spikes appear longer and sometimes curved at higher magnification. The F-actin rings in the bumblebee workers had diameters of 1.99±0.06 µm (*n* = 24), which is about one third smaller than those found in honeybee workers (3.11±0.13 µm, *n* = 8) (compare Fig. 5A and Fig. 5B; see also supplementary material Fig. S3). Ring diameters in the bumblebee did not differ between young and old workers, as well as between workers and queens. However, ring diameters were slightly, but significantly smaller in males (1.69±0.08 µm, *n* = 17) (supplementary material Fig. S3).

**Fig. 6.**
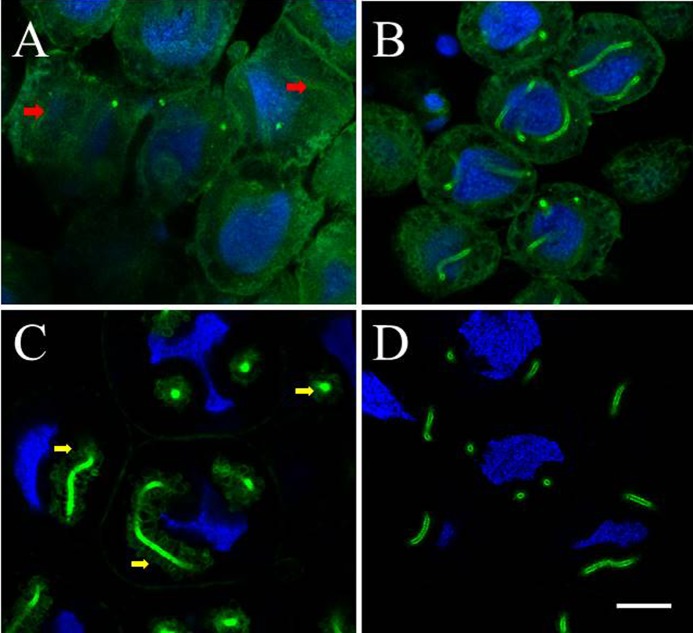
Changes of F-actin organization in the end apparatus and the nuclear morphology of secretory cells. Hypopharyngeal glands of bumblebee workers from different pupal stages ((A) P5 stage, dark eyes, light cuticle; (B) P6/P7, dark eyes and cuticle), and from young adults ((C) <2 hours post eclosion; (D) ∼8 hours post eclosion) were dissected, treated with Hoechst stain (blue) and fluorescently labeled phalloidin (green). (A) The F-actin tubes of the end apparatus begin to arise (red arrows), nuclei are largely round-shaped. (B) More advanced stage of actin tubes formation, septum membrane not delivered, nuclei still round. (C) Septum membrane delivered by directed exocytosis, freshly secreted septal cuticle solidifies, microvilli of the septum held in shape by cortical F-actin (yellow arrows), nuclei become deformed. (D) Septal cuticle solidified, underlying cortical F-actin depolymerizes, shapes of nuclei remain deformed. All figures are at the same magnification. Scale bar: 20 µm.

Interestingly, the shapes of nuclei of secretory cells were always found to be irregular in both species. We noticed that the vicinity of the EA was often associated with nuclear deformations (Fig. 2A, Fig. 4C, Fig. 6C,D). Apparently, the intracellular tubings of the EA were included in these deformations of the cell nuclei; this was confirmed by 3D reconstructions of the nucleus (supplementary material Fig. S4). We speculate that the BtRJPL-free region surrounding the actin tube may be due to a cuticular septum separating the plasma membrane and the EA (Deseyn and Billen, 2005).

### Novel F-actin structures in hypopharyngeal gland cells of the bumblebee

In samples of freshly eclosed bumblebees we identified additional F-actin surrounded stacks of spheres or ovals accumulating near the actin tube of the EA (Fig. 6). About 6 hours later, such structures were almost completely vanished (*n* = 5), indicating that the presence of these structures near the EA was transient. We checked the F-actin structures at different pupal stages. In HGs of young pupae (approximately P5 with dark eyes, cuticle not melanised; Fig. 6A), we found only dispersed F-actin and regularly shaped nuclei. Only in some cells, initial stages of EA tubes were observed (red arrows). In older pupae (P6/P7, dark eyes, melanized cuticle; Fig. 6B), the F-actin tubes of the EA were formed, but no surrounding actin spheres/ovals could be seen and nuclei were still regularly shaped. The F-actin-covered spheres/ovals appeared, for the first time, in freshly eclosed bumblebees (Fig. 6C) and disappeared only few hours later (Fig. 6D).

### BtRJPL is expressed in bumblebee brains

**Fig. 7.**
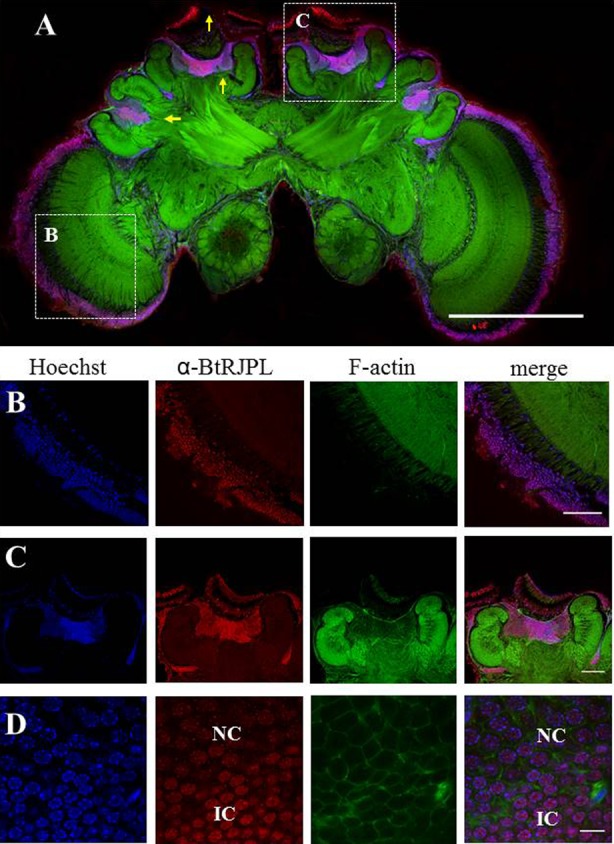
BtRJPL expression in the bumblebee brain. (A) Overview of a frontal section of a worker brain immunolabeled with α-BtRJPL antibodies (red), DNA stain (Hoechst, blue) and phalloidin against the filamentous actin (green). Strong BtRJPL-ir was found in Kenyon cells, cell bodies in the optic lobe and ocelli (yellow arrows). Boxes indicate areas shown in panels B and C (different samples). (B) BtRJPL-ir of cell bodies in the first chiasm of the optic lobes. (C) Detailed view of the median calyx. Both the cell bodies of the inner and clawed Kenyon cells are immunoreactive. (D) Differences in BtRJPL-ir intensity can be seen between inner compact cells (IC) and non-compact cells (NC). Scale bars: 500 µm (A), 100 µm (B,C), 10 µm (D).

**Fig. 8.**
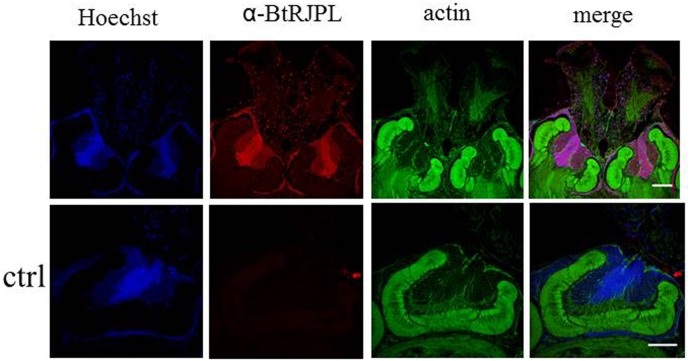
Putative MRJPs in honeybee brains. Sections of honeybee brain were incubated with α-BtRJPL antibodies (red; upper row); in the control the antibody was omitted (lower row). The α-BtRJPL antibody in the honeybee worker MB seems to recognize similar cell bodies as in bumblebees. Scale bars: 100 µm.

We have previously shown that besides the HGs, *BtMRJP* mRNA is also expressed in the brain of bumblebee workers, queens and drones, albeit to a lower extent (Kupke et al., 2012). We therefore set out to detect BtRJPL expression in brain tissues by using immunohistochemical staining of bumblebee brain sections. BtRJPL immunoreactivity (ir) was found in different parts of the brain (Fig. 7A). More precisely, we found distinct labeling in the Kenyon cells, in the outer layer of the ocelli and in the first chiasm of the optic lobes (Fig. 7A–C). Double staining with Hoechst nucleic acid stain revealed that the BtRJPL signal is most likely localized in the cell nuclei (Fig. 7D), but not in axons or dendrites. In addition, some, but not all, nuclei of cells located in the outer layers of the antennal lobes were labeled (supplementary material Fig. S5). Control sections without the α-BtRJPL primary antibody showed no immunoreactivity, indicating the specificity of the antibody staining (Fig. 8; supplementary material Fig. S5). Similar staining patterns were detected in drone and queen brains (not shown), suggesting that the brain localization of BtRJPL is caste- and gender-independent.

### Immunohistochemical detection of MRJPs in honeybee brains

For comparison, we investigated MRJP-ir in honeybee brains by means of the α-BtRJPL antibody. We observed an essentially similar staining pattern compared to bumblebees with the most intensive staining in the central region of Kenyon cell somata in the MB calyces (Fig. 8), the site where *MRJP1* was previously identified by *in situ* hybridization (Kucharski et al., 1998). However, we cannot assign the ir-signal to a particular MRJP protein, since obviously all abundant MRJP proteins bind the α-BtRJPL antibodies with similar affinities (see above; Fig. 1).

## DISCUSSION

### Hypopharyngeal glands are present in all bumblebee castes

Unlike in honeybees, HGs appear to be not restricted to the worker caste in bumblebees. Previously, HGs were described in bumblebee queens, but their presence in males was still a matter of controversy (Palm, 1949; Svensson and Bergström, 1977; Terzo et al., 2007). Here we identified HGs in *B. terrestris* males. We could show that HG tissue is distinct from labial gland tissue at both the morphological and cellular levels (Figs 3, 4). In addition, protein profiles of HGs and LGs differed markedly from each other, and, at the same time, male and female HG profiles appeared to be similar (Fig. 4).

Using specific antibodies, we were able to confirm the expression of BtRJPL in HG cells of all adult castes and sexes. Microscopic observations of bumblebee and honeybee HGs combined with phalloidin-labeling of F-actin and Hoechst labeling of DNA revealed so far unknown or only poorly described morphological features of HG secretory cells. Their differences from secretory cells of honeybee HG will be discussed below.

### Differences in morphology of HG cells and their end apparatus between honeybees and bumblebees

Besides the most remarkable differences in size (∼60 µm in *B. terrestris* and ∼200 µm in *A. mellifera*) and the anatomical features of glandular units (mono- and oligocellular, respectively), major differences were observed in the fine structure of the EA. Honeybee EA contained individual F-actin rings distributed at regular spacing throughout the entire length (Kheyri et al., 2012) (Fig. 5). In contrast, F-actin rings in the bumblebee formed densely packed nearly contiguous stacks of rings forming a tube of about 1/3 smaller diameter compared to that in the honeybee. The morphology of *B. terrestris* EA actin rings resembles roughly these found in *Tetragonula carbonaria* (Kheyri et al., 2012).

Differently shaped HGs and their EA may reflect adaptations to the production of different amounts and physical properties of secretions. Royal jelly, secreted by honeybee HGs, is produced in large amounts and is extremely viscous; it does not drip down from bottom-up oriented queen cells. Wider EA diameter could facilitate movement of this secretion. In fact, supplemental mechanisms may be necessary to move the secreted RJ through EA in the honeybee. However, Kheyri et al. did not find any signs of contractibility of the EA (Kheyri et al., 2012). Little is known about secretion of bumblebee HGs, but its appearance is clear and not particularly viscous (Pereboom, 2000). It contains digestive enzymes such as amylase and invertase and, therefore, most probably participates in food digestion, i.e. saliva-like function (Palm, 1949).

### Actin re-arrangements during postembryonic development of the end apparatus

The two novel actin structures we found in bumblebees, stacks of spheres or ovals and actin “spikes”, may be related to each other. The F-actin spikes extending from the surface of EA (Fig. 2, yellow arrows) may be remnants of actin-covered ovals seen in early stages of HGs development (Fig. 6). We hypothesize that the function of the latter could be in the generation and early maintenance of membrane microvilli. Microvilli are common to many secretory cells including bee HGs, where they form the septum around the EA (Deseyn and Billen, 2005). Usually, the shape of microvilli is stabilized by cortical actin microfilaments beneath the membrane. However, insect cells are covered by an extracellular cuticular exoskeleton, which also fills the cell–EA interface (Kheyri et al., 2012; Noirot and Quennedey, 1974). Absence of F-actin underneath microvilli of older animals may indicate that the cuticle took over the role of microvilli shaping. However, cuticle needs to be secreted and requires some time to harden (Moussian, 2010). Thus the process of microvilli formation and shaping in a period immediately after eclosion, before cuticle solidifies, may be supported by actin filaments. Once the cuticle becomes solid, F-actin may depolymerize (Fig. 6C,D). Our hypothesis contradicts the common view claiming that EA is formed by invagination of the cytoplasmic membrane (Beams et al., 1959; Cruz-Landim, 1998; Painter and Biesele, 1966). It explains how an intracellular structure, such as the recently found F-actin tube of the EA (Kheyri et al., 2012), which is in fact located extracellularly, makes its way out of the cytosol. Accordingly, the actin tube is initially formed in the cytosol and later isolated from the cytosol by directed exocytosis and fusion of the secretory vesicles forming the septum membrane. Later, septum membrane microvilli are formed and secreted cuticle solidifies and fixes them, forming a mature EA and surrounding septum.

Rigid tubings of the EA and surrounding septum appear to represent a mechanical hindrance affecting morphology of intracellular organelles of secretory cells. This is best documented by the deformed shape of the nuclei, which always notch in the proximity of the EA (Fig. 4C, Fig. 6; supplementary material Fig. S4). It is difficult to conceive that such deformed nuclei could condense chromosomes near their central plane and enter mitosis. Also further phases of mitosis characterized by regularly arranged chromosomes (i.e. in the metaphase) and their concerted separation between daughter cells (telophase) would be problematic due to rigid tubes in the cytosol.

In agreement with the statement above, we did not find a single mitotic cell among thousands of HG secretory cells inspected in the course of our study. However, it is known that foraging honeybee workers can reverse their behavior from foraging back to nursing, when nurses are scarce in the colony. During this process they restore fully functional HGs (Hrassnigg and Crailsheim, 1998; Maleszka et al., 2009; Ohashi et al., 2000). It would be interesting to investigate in future studies whether the F-actin system undergoes remodeling during this process.

### MRJPs in the brain

Besides MRJPs expression in the honeybee HGs, several independent studies reported on MRJP expression in the brain (Hernández et al., 2012; Hojo et al., 2010; Kucharski et al., 1998; Peixoto et al., 2009). In particular, selective *MRJP1* expression in Kenyon cells of the mushroom bodies (MBs) may indicate an important non-nutritive function since the MBs were shown to be associated with sensory integration and learning and memory in bees (Hourcade et al., 2010; Komischke et al., 2005; Menzel, 2001). A single-copy MRJP-like protein in bumblebees, which possibly represents an ancestral state of MRJP evolution (Kupke et al., 2012), is an ideal candidate to ask for the original function and localization of the MRJP in both secretory tissues and in the brain.

Here we could show that the expression of BtRJPL occurs in both HGs and in Kenyon cells of the brain, indicating that this type of expression pattern is not honeybee specific but may represent an original rather than a derived state. Moreover, distinct localization of BtRJPL in the inner compact Kenyon cells correlates with the finding of Kucharski et al. obtained by in situ hybridization (Kucharski et al., 1998). There are several pieces of circumstantial evidence supporting BtRJPL expression in the brain: (1) *BtRJPL* mRNA was detected by RT-qPCR in bumblebee brains (Kupke et al., 2012), (2) at least one of the honeybee homologs of BtRJPL, MRJP1, was detected by *in situ* hybridization to be localized in the Kenyon cell bodies (Kucharski et al., 1998), and (3) similar neurons (predominantly inner compact Kenyon cells) were labeled by the anti-BtRJPL antibody in both *B. terrestris* and *A. mellifera* brains (compare Figs 7 and 8).

The obviously multiple functions of MRJP proteins in bees suggested by the diverse expression pattern are not uncommon. For example, the oldest and most characterized protein of the Yellow/MRJP protein family, Yellow, which is part of the insect cuticle pigmentation, is also synthesized in *Drosophila* brains, where it decisively regulates the courtship behavior of males (Radovic et al., 2002). Currently, we can only speculate about a potential function of MRJP proteins in the bee brain. One possibility might be a function as a growth factor involved in the growth or plasticity of Kenyon cells. Growth factor-like activity of MRJP1 in worker/queen switch, documented in detail by Kamakura (Kamakura, 2011), goes along this way. Another possibility may reside in the intrinsic capability of several proteins belonging to the Yellow/MRJP family to bind and modify biogenic amines such as DOPA and dopamine (Han et al., 2002; Xu et al., 2011). Since dopaminergic neuromodulation and -transmission is common in insect brains (Blenau and Erber, 1998), a direct or indirect involvement of neuronal MRJPs in this type of neuronal communication is possible, but further studies are necessary to test these ideas.

### Conclusions

By employing immunohistochemistry we could show that the general appearance, cellular and subcellular structure of HGs differs substantially between honeybees and bumblebees. Whereas honeybee HGs are absent in males and queens, all castes and sexes in bumblebees possess HGs, albeit, male HGs are much smaller. We conclude that HGs may have evolved from universal caste-independent glands to food-producing glands in honeybee workers. Furthermore, we could show that the MRJP-like protein of bumblebees is synthesized, besides the hypopharyngeal glands (HGs), in somata of certain neuronal cells, predominantly in the inner compact Kenyon cells of the mushroom bodies, centers for learning and memory in the insect brain. Our findings implicate multiple functions of the MRJP in the brain and HGs of bumblebees.

### List of abbreviations

BtRJPL, *Bombus terrestris* royal jelly protein-like; DOPA, L-3,4-dihydroxyphenylalanine; EA, end apparatus; F-actin, filamentous actin; HGs, hypopharyngeal glands; ir, immunoreactivity; LGs, labial glands; MRJP, major royal jelly protein; NGS, normal goat serum; OC, ocelli; PBS, phosphate-buffered saline; RJ, royal jelly; SDS-PAGE, sodium dodecylsulphate polyacrylamide gel electrophoresis; TBS, Tris-buffered saline; TBST, Tris-buffered saline with 0.05% Tween-20.

## Supplementary Material

Supplementary Material
