# Supplementary material for: Royal jelly-like protein localization reveals differences in hypopharyngeal glands buildup and conserved expression pattern in brains of bumblebees and honeybees
Source: Biol Open. 2014 Mar 25;3(4):281–8. doi: 10.1242/bio.20147211 (PMC3988797; doi:10.1242/bio.20147211)
Supplement: Supplementary Material [file supp_3_4_281__index.html]

Royal jelly-like protein localization reveals differences in hypopharyngeal glands buildup and conserved expression pattern in brains of bumblebees and honeybees — Supplementary Material 

# Royal jelly-like protein localization reveals differences in hypopharyngeal glands buildup and conserved expression pattern in brains of bumblebees and honeybees

## bio.20147211 Supplementary Material

**Files in this Data Supplement:**

- Supplementary Material - štefan Albert et al. doi: 10.1242/bio.20147211
