# Supplementary material for: Royal jelly-like protein localization reveals differences in hypopharyngeal glands buildup and conserved expression pattern in brains of bumblebees and honeybees
Source: Biol Open. 2014 Mar 25;3(4):281–8. doi: 10.1242/bio.20147211 (PMC3988797; doi:10.1242/bio.20147211)
Supplement: Supplementary Material [file supp_bio.20147211_bio.20147211-s1.pdf]

Biology Open (2014) 000, 1–8 doi:10.1242/bio.20147211

Supplementary Material

Štefan Albert et al. doi: 10.1242/bio.20147211

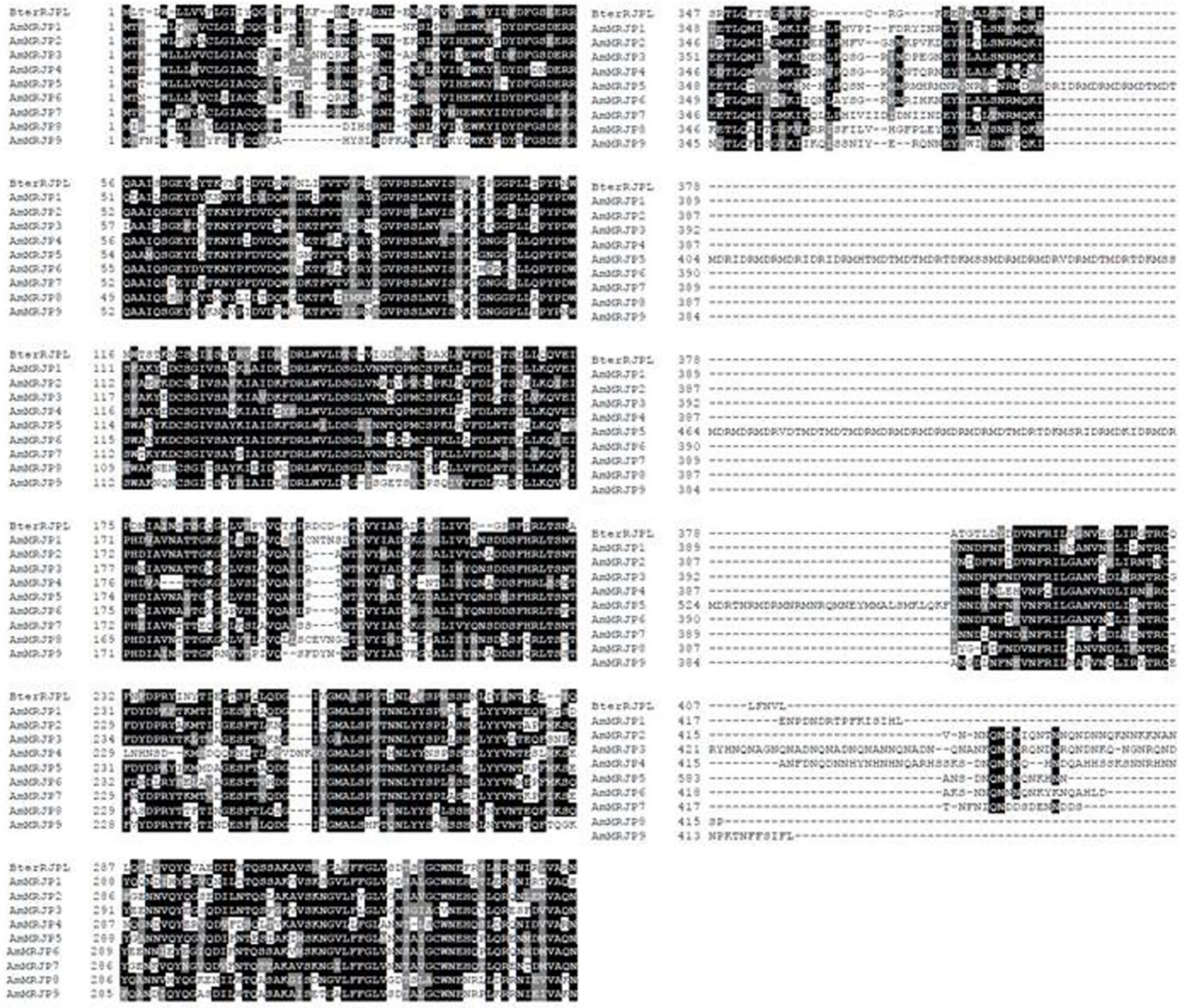

Fig. S1. Alignment of bumblebee BtRJPL and honeybee MRJPs. The protein sequences were aligned using CLUSTAL W and visualized by BOXSHADE. Black-shaded residues are identical among majority of sequences, grey-shading indicates substitution by chemically similar amino acid.

Biology Open

Biology Open (2014) 000, 1–8 doi:10.1242/bio.20147211

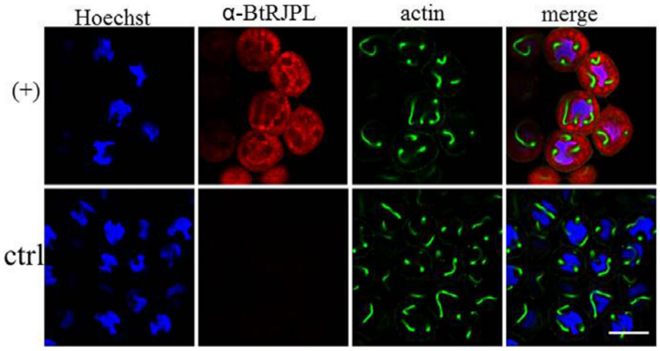

Fig. S2. BtRJPL-ir of hypopharyngeal glands. The antibody stains proteins in the cytosol and in the center of the end apparatus tubes (red spots in the transversal sections of the end apparatus), but is absent in the septum surrounding the end apparatus (green tubes). Note that the nuclei are deformed in the vicinity of the end apparatus. (+), incubation with anti-BtRJPL antibodies; ctrl, antibody omitted. Scale bar: 50 µm.

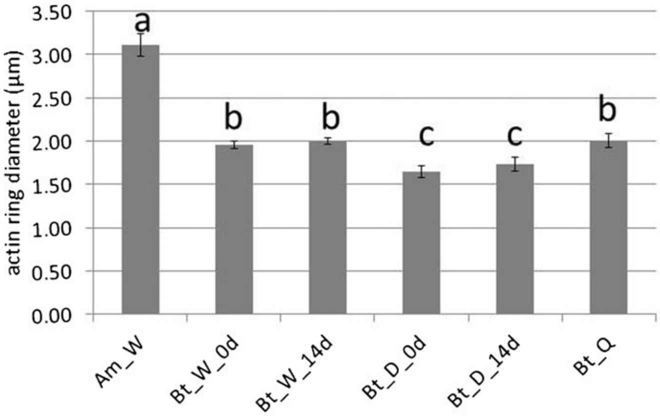

Fig. S3. F-actin ring diameters of the end apparatus differ between females and males in *B. terrestris* and between bumblebees and honeybees. Diameters between groups marked with the same letter did not differ, those between groups were significantly different (Mann–Whitney U-test, $p<0.001$). Am_W, *A. mellifera* workers; Bt_W, *B. terrestris* workers; Bt_D, *B. terrestris* males; Bt_Q, *B. terrestris* queen; 0 d, freshly eclosed; 14 d, 14 days old.

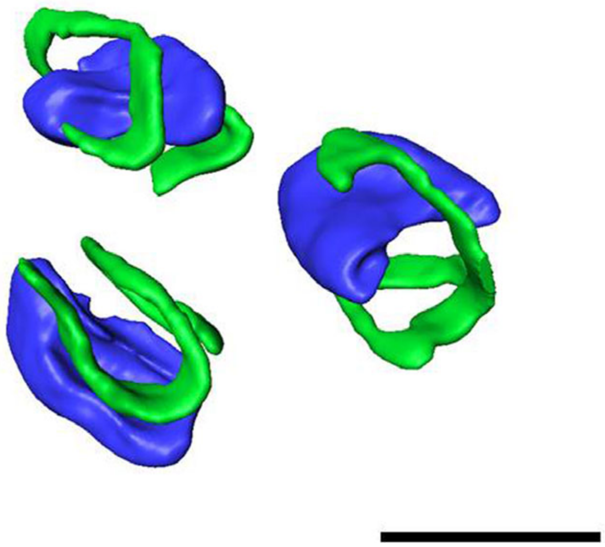

Fig. S4. 3-dimensional reconstruction of nuclear shapes (blue) in the vicinity of the end apparatus (green). Serial optical sections of the sample were photographed and used for 3-D reconstruction by means of AMIRA software (for details, see Materials and Methods). Scale bar: 50 µm.

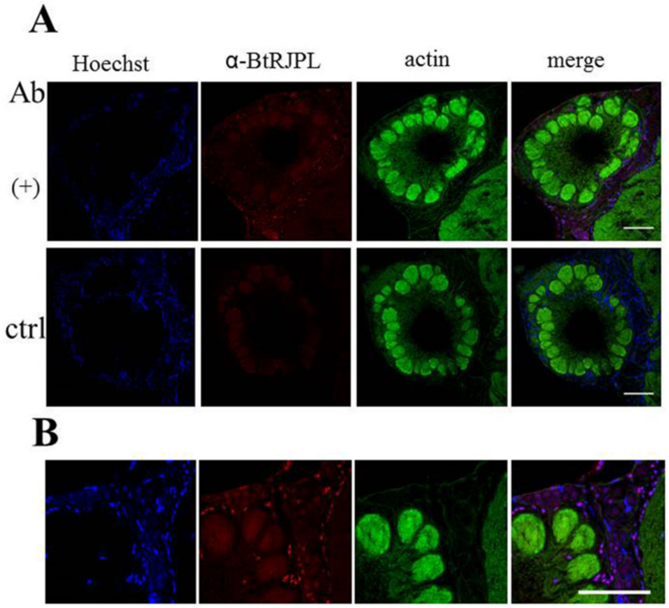

Fig. S5. BtRJPL-ir of a bumblebee worker's antennal lobe. (A) In contrast to mushroom body calyces, overall labeling is weaker (upper row); however, in the control no signal can be seen (lower row). (B) Only a subset of cell somata adjacent to the glomeruli is labeled. (+), incubation with anti-BtRJPL antibodies; ctrl, primary antibody omitted. Scale bars: 100 µm.

Biology Open
